# Biographical Feature: In memoriam Pierre Tiollais (1934–2024)

**DOI:** 10.1128/jvi.02125-24

**Published:** 2025-01-16

**Authors:** Christian Bréchot, Patrick Charnay, Hugues de Thé, Anne Dejean, Marie-Louise Michel, Pascal Pineau, Pierre Sonigo, Simon Wain-Hobson, Yu Wei, Jean Weissenbach

**Affiliations:** 1 Global Virus Network, University of South Florida 7831 , Tampa, Florida, USA; 2 Ecole Normale Supérieure, PSL Research University, CNRS, INSERM, Institut de Biologie de l’Ecole Normale Supérieure (IBENS) 206947 , Paris, France; 3 Collège de France, Inserm, CNRS, PSL Research University, University Paris Cité, AP/HP: St-Louis Hospital 55663 , Paris, France; 4 Inserm U933, Paris, France; 5 Institut Pasteur 27058 , Paris, France; 6 Sebia, Lisses, France; 7 Génomique Métabolique, Genoscope, Institut François Jacob, CEA, CNRS, University of Évry, Université Paris-Saclay 27048 , Evry, France; The University of Arizona, Tucson, Arizona, USA

**Keywords:** hepatitis B virus, vaccine

## TEXT

**Figure:**
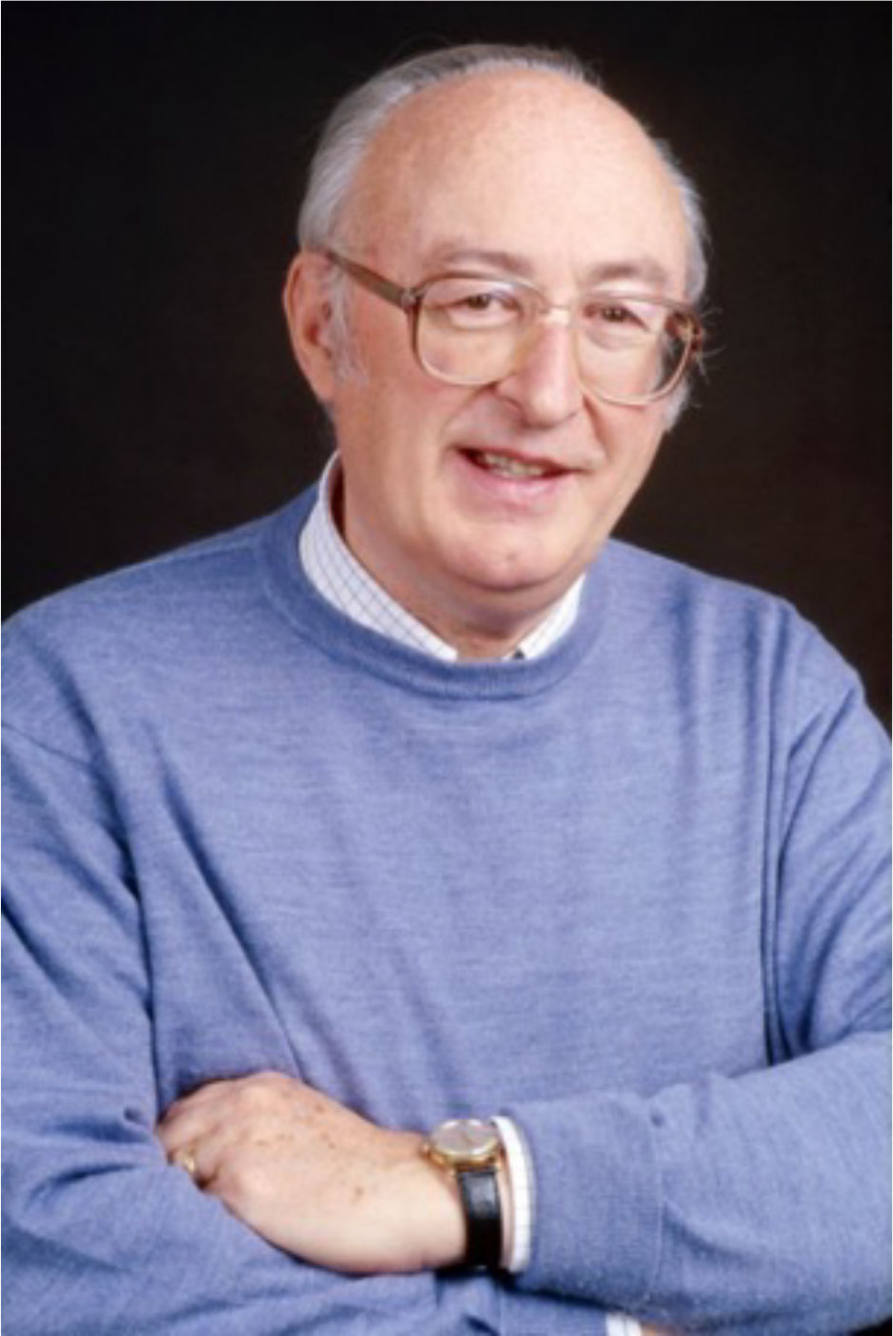


Pierre Tiollais, a French physician-biologist who cloned and sequenced the genome of hepatitis B virus (HBV) and developed a recombinant hepatitis B vaccine, died on 5 August 2024, at the age of 89. Pierre had his wife Irène and his son Romain close by his side through the difficult times since he had been ill. Born on 8 December 1934 in Rennes, Brittany, to pharmacist parents, Pierre remained attached to his native province, which in France is characterized by a strong anthropological particularism, throughout his life and always proudly defined himself as a Breton.

Pierre began his medical studies in Rennes and completed his residency in hematology, obtaining an M.D. from the University of Paris in 1968. Pierre became interested in biology after he took a break from his career as a medical doctor to enroll in the Faculty of Biology at Jussieu (now Sorbonne University) and learn biochemistry, a discipline he would love for the rest of his life. Pierre began his scientific career at INSERM (French National Institute of Health and Medical Research) in 1967 and joined the Pasteur Institute in Paris in 1973. Convinced that molecular biology and genetic engineering, then booming, were the key to future research, Pierre went to Cold Spring Harbor Laboratory in the early 1970s to learn how to produce and use restriction enzymes. He became a specialist in recombinant DNA technologies, developing the first cloning vector derived from the lambda bacteriophage virus in 1975.

With his training in medicine, Pierre was keen to apply basic research to public health issues. At the suggestion of his residency mentor Jean Bernard at the Saint-Louis Hospital, Pierre turned to hepatitis B virus, whose genome was then unknown. The story told is that knowing that Baruch Blumberg would be in transit in Paris, Pierre went to Charles de Gaulle airport to meet the discoverer of HBV. As usual, Pierre worked methodically and meticulously on his new research topics. Having an acute awareness of the need for the most modern facilities, Pierre learned the set-up of a high-security biosafety level 3 (BSL-3) facility at Uppsala University, Sweden, and built the first BSL-3 laboratory in France at Pasteur Institute. His savoir-faire in recombinant DNA technologies greatly advanced the pace of HBV genome cloning and sequencing in a context of international competition, publishing with Francis Galibert the first complete HBV genome in 1979 (1). In the same year, Pierre was appointed chief of the Genetic Recombination and Expression Unit at Pasteur Institute, a joint laboratory of Pasteur Institute, INSERM, and CNRS (French National Centre for Scientific Research). In the early days of recombinant DNA, Pasteur Institute and Pierre Tiollais’ laboratory, in particular, were frequently called by other investigators to help them in their cloning projects. Such invaluable help has been to the benefit of science in general.

The following year, Pierre’s laboratory made discoveries of the presence of integrated HBV sequences in cellular DNA of human hepatocellular carcinoma (HCC) (2), providing a key argument for the involvement of HBV in the etiology of liver cancer. Later, his laboratory demonstrated that oncogenic activation of cellular genes by integrated HBV sequences *in cis* contributed to hepatocyte transformation (3, 4), giving rise to the hypothesis of insertional mutagenesis by the virus (5). This hypothesis was particularly validated in Pierre’s laboratory in woodchuck hepatitis B virus (WHV)-induced HCC, in which insertional mutagenesis of *myc* family genes by WHV was found in more than 80% of tumors (6–8).

Less known, but not less significant, Pierre participated in the cloning and sequencing of HIV and simian immunodeficiency virus at Pasteur Institute during the early outbreak of AIDS (9, 10), making contributions to the understanding of molecular biology of this newly identified infectious agent that was threatening the humanity.

While studying molecular biology of viruses, Pierre did not lose sight of public health and the improvements that could be made to it with his research. Following the cloning of HBV genome, he and his team were devoted to the development of a recombinant vaccine against hepatitis B. While groups in the United States developed hepatitis B vaccine expressed in yeast cells, Pierre’s team chose to produce HBsAg 22 nm particles in Chinese hamster ovary (CHO) cells for vaccine candidate (11). In collaboration with David Milich and Francis Chisari of Scripps Clinic and Research Foundation, La Jolla, California, they showed that the HBsAg 22 nm particles obtained from CHO cells, which contained not only the small protein but also the middle protein of HBV envelope, are highly immunogenic in mice, paving the way for clinical development of the vaccine (12). The recombinant hepatitis B vaccines represent the first examples of the application of genetic engineering to human vaccination. Since the recombinant hepatitis B vaccine came to market in 1986, massive vaccination campaigns have been taking place in endemic regions in the world. By one estimation, in 98 low- and middle-income countries, 38 million deaths will be prevented due to hepatitis B vaccination among people born between 2000 and 2030 (13). Pierre was always proud to present that hepatitis B vaccines had saved lives.

Pierre possessed a sense of community and a moral commitment to sharing scientific advancement. He was one of the early organizers of the International HBV Meeting, which is still held annually. Pierre frequently visited HBV-endemic regions in Africa and South-East Asia. He was the best-known ambassador of genetic engineering, HBV biology, and liver cancer in China, a country with the highest number of HBV chronic carriers. His collaboration and friendship with Zaiping Li and Yuan Wang of Shanghai Institute of Biochemistry and Cell Biology, Chinese Academy of Sciences, had lasted for four decades.

Pierre was appointed a full professor at Pasteur Institute in 1988 and professor of exceptional class at the Medical School Lariboisière-Saint-Louis, University Paris 7, in 1991. He was the recipient of numerous prestigious prizes, including the prize of the Athéna Foundation—Institut de France, the grand prix of the Foundation for Medical Research, and the grand prix of Institut Electricity Health. He was elected member of the French Academy of Sciences in 1991, the French Academy of Medicine in 1996, and Chinese Academy of Engineering in 1998.

Throughout his life, Pierre valued informality, imbuing his laboratory with complete autonomy to his younger collaborators. He used to say “if a scientist wants to paint a wall in green, let him paint it in green.” Some of these younger collaborators greatly benefited from the umbrella of his lab and the vivid scientific atmosphere before their settlement in a new remote environment. In his laboratory, fields as differing as adenovirus (14), interferon beta (15), HIV, retinoic acid receptor, and human molecular genetics shared one roof with HBV. His laboratory had produced so many scientific leaders in France that it formed the so-called “Tiollais School.” Pierre considered it as one of his major achievements in his career.

Those who had the privilege of working with Pierre will remember him as a generous, unassuming, and warmhearted mentor. We will miss him.
